# Xylanase Impact beyond Performance: Effects on Gut Structure, Faecal Volatile Fatty Acid Content and Ammonia Emissions in Weaned Piglets Fed Diets Containing Fibrous Ingredients

**DOI:** 10.3390/ani12213043

**Published:** 2022-11-05

**Authors:** Waewaree Boontiam, Pheeraphong Phaenghairee, Veerle Van Hoeck, Bindhu Lakshmibai Vasanthakumari, Ingrid Somers, Alexandra Wealleans

**Affiliations:** 1 Faculty of Agriculture, Division of Animal Science, Khon Kaen University, Khon Kaen 40002, Thailand; 2 Kemin Europa N.V., Animal Nutrition and Health EMENA, Toekomstlaan 42, 2200 Herentals, Belgium; 3 Kemin Industries, 1900 Scott Avenue, Des Moines, IA 50317, USA

**Keywords:** non-starch polysaccharides, exogenous enzyme, piglets, nutrient availability, growth performance, intestinal health

## Abstract

**Simple Summary:**

The use of fibrous dietary ingredients can improve the profitability of pig production but can have detrimental effects on nutrient digestibility. To improve the utilisation of dietary fibre in pigs, this study aimed to evaluate the impact of increasing levels of xylanase supplementation on growth performance, nutrient digestibility, gut morphology and faecal volatile fatty acid and ammonia concentrations in weaning pigs. Xylanase significantly improved growth and nutrient digestibility, while also changing gut structure and metabolite levels, perhaps suggesting an underlying shift in intestinal microbiota.

**Abstract:**

The addition of xylanase to piglet diets is known to improve performance and nutrient digestibility. The present study aimed to assess the impact of new xylanase on the growth performance, nutrient digestibility, and gut function of weaned piglets. A total of 144 pigs, weaned at 28 days (7.48 kg initial body weight, IBW), were assigned to 36 pens and 9 pens per treatment. Dietary treatments were a basal complex control diet, and the basal diet supplemented with 45,000, 90,000 and 135,000 U/kg xylanase. Performance was measured at days 0, 14 and 35. At day 35, samples were collected for assessment of intestinal histology, and volatile fatty acid and ammonia concentrations. After two weeks post-weaning, additional 12 piglets (11.34 kg IBW) were placed in metabolic crates for assessment of apparent total tract nutrient digestibility using a dietary marker. The addition of xylanase at 90,000 and 135,000 U/kg significantly improved average daily gain (333.6 g/day control, 364.86 g/day, 90,000 U/kg, 405.89 g/day, 135,000 U/kg, *p* < 0.05), G:F (0.557 control, 0.612 90,000 U/kg, 0.692 135,000 U/kg, *p* < 0.05), and reduced diarrhoea. This was driven improved nutrient digestibility and villus height in the jejunum (372.87 µm control, 432.53 µm 45,000 U/kg, 465.80 µm 90,000 U/kg, 491.28 µm 135,000 U/kg, *p* < 0.05). Xylanase supplementation also linearly increased faecal butyrate levels and had a quadratic relationship with propionate concentrations. 135,000 U/kg xylanase also reduced ammonia emissions. In conclusion, dietary supplementation with xylanase improved growth performance and feed efficiency in weaning piglets, likely driven by improvements to gut structure and function.

## 1. Introduction

Increasing levels of non-starch polysaccharides (NSPs) in piglet diets, negatively affect nutrient digestibility and growth performance, as the enzymes produced are not efficient to degrade some fibre components [1,2,3]. This problem is particularly evidenced in weaning diets, when the combined stressors of removal from the sow, social mixing, and new feed sources and method of acquisition cause the production of endogenous enzymes and stomach acid to drop [4,5]. High-fibre diets can also reduce feed intake, and thereby insufficient energy and nutrient utilisation to support growth performance [6,7]. By contrast, low-fibre diets are linked to numerous detrimental effects on gut health including thinning of the mucus layer, increasing susceptibility to infection, and induction of dysbiosis and probiotic extinction in the gut [8].

To improve nutrient digestibility and performance, commercial nutritionists rely on the addition of exogenous enzymes, including xylanase. Xylanase degrades arabinoxylan, the main NSP found in the major cereals used in animal feed [9]. Addition of xylanase to piglet diets has shown to reduce intestinal viscosity [10,11], increase nutrient digestibility [1,12] and improve performance [1,10]. Meta-analysis of xylanase’s impact on pig growth performance suggests that responses can be inconsistent between studies [13,14]. The size of the effect is influenced by several factors, including the specific xylanase molecule tested [15] and the composition of the basal diet [16,17], among others.

It is thought that this is due to the interaction of xylanase and the gut environment and intestinal microbiota. By breaking down long arabinoxylan chains into shorter arabinoxylan-oligosaccharides (AXOS), xylanase addition changes the availability of substrates for microbiota growth [17]. This favours beneficial *Lactobacillus*, *Ruminococcus*, *Prevotella* and *Bifidobacteria* populations and reduces potentially pathogenic *Clostridia* and *Pasteurella* counts [8,18,19,20,21,22]. These bacteria ferment NSP into short-chain fatty acids, which are used in “cross-talk” feedback loops that encourage the proliferation of other, associated beneficial bacteria. These shifting populations subsequently augment the barrier function of the host intestine [23], allowing better nutrient absorption and disease resilience.

These changes in bacterial populations, combined with the traditional reductions in viscosity, drive a healthier, better functioning gut environment with longer villi and larger absorptive surfaces [10,24], increased digestibility and absorption, and finally improved growth performance. Effects of xylanase supplementation on gaseous emissions are less clear: Kpogo et al. [25] saw no effect of a multi-enzyme blend on faecal gas emissions, and similarly, Chen et al. [26] found that a multi-enzyme blend had no significant effect on NH_3_ levels, though significant reductions were seen in CO_2_ production.

The link between xylanase supplementation, improved gut health and function and improved post-weaning performance is well established. The current experiment was designed to study the effects of increasing levels of xylanase supplementation in weaned piglets fed complex diets with the inclusion of fibrous ingredients on the production performance, nutrient digestibility, intestinal tract morphology and volatile fatty acid concentration in faeces.

## 2. Materials and Methods

Two experiments were conducted to assess the effect of xylanase supplementation: a pen trial to assess growth performance and a metabolic trial to assess nutrient retention. Both studies took place in a commercial pig farm in Nakhon Pathom province, Thailand. All experimental procedures followed the guidelines of the National Research Council of Thailand and were approved by the Animal Care and Use Committee of Khon Kaen University (permission No. IACUC-KKU79/63).

### 2.1. Experimental Diets

The same diets were used for both studies. A two-phase feeding program was used: a pre-starter feed from day 1 to day 14 and a starter feed from day 15 to day 35. Basal diets were formulated using corn, wheat, and soybean meal. All nutrient requirements of each experimental diet were calculated to meet or exceed the recommendation of NRC [27], presented in Table 1. All diets were manufactured before the onset of the experiment. The experimental pellet feeds (4 mm in both phases) were prepared at 75 °C and were manufactured at the feed mill of Bangkok Animal Research Center Co., Ltd. (Samut Prakan, Thailand; #AF20/28A). Feed samples (500 g/treatment) were immediately delivered to Kemin Europa N.V. (Herentals, Belgium) for xylanase recovery as described by Van Hoeck et al. [18].

There were four dietary treatments: (1) a control diet, (2) control diet supplemented with 45,000 U/kg xylanase, (3) control diet supplemented with 90,000 U/kg xylanase, and finally (4) control diet supplemented with 135,000 U/kg xylanase. The xylanase used in the study was an intrinsically thermostable, monocomponent xylanase produced by *Thermopolyspora flexuosa* expressed in *Pichia pastoris* (Xygest^TM^ HT, Kemin Animal Nutrition and Health, Herentals, Belgium), and is a beta 1–4, an endo-xylanase enzyme belonging to the GH11 family.

### 2.2. Experiment 1—Performance Study

A total of 144 weaned pigs ([Large white × Landrace] × Duroc; 7.48 ± 0.24 kg initial BW) weaned at day 28 were used in a 5-week growth trial. Upon arrival, piglets were randomly allotted to one of four dietary treatments in a randomised complete block design. Treatments were replicated in nine pens, with four piglets (two gilts and two barrows) per pen.

During the experiment, all pigs were housed in an environmentally controlled building with half-slatted concrete floors (0.96 m × 2.16 m) with a stocking density of 0.52 m^2^ per pig. The pen was equipped with heating using an electric bulb, feeder and nipple drinker to provide water and feed with ad libitum access throughout the study. Unused rice straw was provided for two weeks postweaning to minimise temperature stress. The housing temperature was maintained at 30 °C for the first week of the study, after which it was gradually reduced by 1 °C every week. Microclimate conditions (temperature and relative humidity) were recorded daily.

Pig body weight and feed intake were recorded on weeks 0, 2, and 5 post-weaning, and used for evaluating average daily gain (ADG) and average daily feed intake (ADFI) observations. ADG and ADFI were calculated by dividing the total weight gain and total feed intake per pen by the total number of experimental days. The gain-to-feed ratio (G:F) was calculated for each pig by dividing the ADG by the ADFI. Mortality was registered when occurred (the date and weight of dead pigs were recorded). The number of diarrheic piglets was recorded daily at 7:00 a.m. to calculate the diarrhoea rate. 

A visual assessment of diarrhoea occurrence was performed every morning at 0800. The four consistency categories were: score 0 = firm and shaped, score 1 = soft and shaped, score 2 = loose and score, and 3 = watery, where scores of 0 and 1 indicate normal faeces and scores of 2 and 3 indicate diarrhoea. The diarrheal rate (%) was calculated as number of piglets with diarrheal/(total number of piglets × diarrhoea days) × 100.

### 2.3. Experiment 1—Intestinal Morphology and Volatile Fatty Acid and Ammonia Concentrations

On day 35, a total of 28 pigs (seven pigs per treatment), having the average pen BW were selected for euthanasia with an overdose injection of barbiturate (200 mg/kg BW). The tissue samples of duodenum and jejunum were flushed with a 0.85% saline solution and fixed in 10% formaldehyde solution for 18 h and then transferred to 70% (*v*/*v*) ethanol until performing the analyses. Fixed intestinal samples were embedded in paraffin. Each fixed sample was sectioned into 5-µm slices by microtome and stained with Harris’ Alum haematoxylin and counterstained with eosin following standard procedures. Villus height (VH) and crypt depth (CD) were assessed using Scion image software (Scion Corporation, Frederick, MD, USA). The VH was obtained from the tip of the villi distance to the villus-crypt junction. The CD was the invaginated depth between the adjacent villi and the villus width. The values recorded from 10 intact, well-oriented longitudinally crypts were chosen in duplicate per each intestinal segment and were then represented as the average value. The villus height/crypt depth ratio (VH: CD ratio) was computed from the measurements above.

For assessment of volatile fatty acid (VFA) contents, faecal samples were taken from 28 euthanised pigs (seven samples per treatment) at the end of the experiment. VFA concentrations were determined after metaphosphoric acid derivation. Briefly, about 0.2 g of faeces was mixed with 0.2 mL of distilled water for 10 min, prior to centrifuge at 1872× *g* for 30 min. The supernatant (0.2 mL) was vortexed with 40 μL in a 25% metaphosphoric acid solution for 10 min, stored at −20 °C for 12 h, and centrifuged at 12,000× *g* for 10 min. The solution of 4-methyvaleric acid (0.1 mL, catalogue # SHBL3457) was an internal standard to quantify VFA concentration using a gas chromatograph (7890A Agilent Technologies, Santa Clara, CA, USA), as followed by the manufacturer’s protocol. The temperature for the injector-port and flame ionisation detector were 230 °C and 250 °C, respectively. The initial temperature was maintained at 120 °C for 4 min prior to injection and gradually increased at 4 °C/min to 160 °C, as helium was a carrier gas.

For assessment of ammonia emissions, fresh faecal samples were collected at the end of the experiment by direct rectal massage of 32 pigs (eight samples per treatment). A total of 50 g of fresh faecal samples were collected, kept in a 2.6-L sealed plastic box, and then delivered directly to a commercial laboratory (Betagro Science Centre, Pathum Thani, Thailand) for subsequent analysis. The samples were incubated at room temperature for seven days to allow for fermentation using the method described by Cho et al. (2008). A total of 100 mL of headspace air was sampled for ammonia quantification using a gas detector (4 LK Detector tube; Gastec Corp., Kanagawa, Japan).

### 2.4. Experiment 2—Nutrient Digestibility

For evaluating nutrient total tract digestibility, a total of 12 male piglets (11.34 ± 0.67 kg initial BW) reared alongside those of Experiment 1 were randomly selected and assigned to four treatments following a completely randomised design for a 6-day adaption period and five days of collection. Prior to entry into the metabolism crates, pigs had been fed a commercial diet. All pigs were housed in individual metabolic crates (0.85 m × 1.15 m × 0.68 m) at a controlled temperature of 27 °C. The experimental diets, identical to the starter diets of Experiment 1, were provided twice daily at 0700 and 1900 according to the rate of 2.0 times the maintenance requirement for ME [27] based on initial BW, with ad libitum access to water. Chromic oxide and ferric oxide (5 g/100 g of feed) were mixed in each experimental diet as an indigestible marker at the beginning and end meal, respectively. Faeces collection was determined using the marker-to-marker approach. Total excreta and urine were individually collected during a collecting period and then kept at −20 °C until analyses. The faecal samples were dried at 60 °C for 72 h. Then, the representative samples were ground (2 mm screen, Wiley mill, Swedesbory, NJ, USA) using a centrifugal mill for later chemical analysis.

Homogeneous samples of feed and faeces were analysed in duplicate to determine the nutrient digestibility of organic matter (OM, method # 930.15), crude protein (CP, method # 984.13; N × 6.25), ether extract (EE, method 920.39), crude fibre (CF), acid detergent fibre (ADF) and starch following the protocol of AOAC [28] and Van Soest et al. [29]. Gross energy was determined using an adiabatic oxygen calorimeter. Neutral detergent fibre (NDF) was defined using an Ankom fibre analyser (Ankom Technology, Macedon, NY, USA). Apparent total tract digestibility (ATTD) was calculated as the following equation:

$$X_{\mathrm{apparent}\;\mathrm{digestibility}}=\frac{X_{\mathrm{ingested}}-X_{\mathrm{excreted}}}{X_{\mathrm{ingested}}}\times 100$$

where X represents OM, DM, CP, EE, CF, and ADF. Urinary nitrogen was quantified, as per method #984.13 [30].

### 2.5. Statistical Analysis

Data were analysed in the Fit Model platform of JMP 15, with treatment as the fixed effect. The experimental unit was the sample replicate or individual pen. Means separation was conducted using Tukey’s HSD. To assess the linear and quadratic effects of increasing the xylanase dose, a second model was run using U/kg and U/kg squared as model effects. Significance was determined at *p* < 0.05; *p* < 0.1 was taken to indicate a near-significant trend.

## 3. Results

Analysed enzyme activities in feed samples are shown in Table 2.

### 3.1. Growth Performance and Diarrhoea Incidence

The effects of xylanase supplementation on the growth performance of weaned piglets are shown in Table 3. In the pre-starter diets, ADG and G:F were linearly improved by increasing the inclusion level of xylanase (*p* < 0.01). The ADG was increased by 24.5% (285.69 g/day XYL vs. 229.52 g/day CON) and G:F was increased by 29.8% (0.810 g gain/g feed XYL vs. 0.624 g gain/g feed CON). In the starter and overall periods, there were linear improvements in ADG, ADFI, and G:F ratio as the XYL increased (*p* < 0.01). At day 35, BW was significantly increased by supplementation with xylanase inclusion in a linear response (21.67 kg XYL vs. 19.14 kg CON, +13.2%, *p* < 0.001). However, significant quadratic relationships were only seen for ADFI in the starter phase (*p* = 0.0182) and across the whole study (*p* = 0.0156). The XYL addition also reduced the diarrhoea rate, with the control suffering 15.22%, compared to, respectively, 9.78%, 7.89%, and 5.89% in the 45,000, 90,000 and 135,000 U/kg groups.

### 3.2. Nutrient Digestibility and Nitrogen Balance

Nutrient digestibility response to xylanase supplementation is summarised in Table 4. Xylanase supplementation linearly increased the digestibility of all measured nutrients except crude fibre (*p* < 0.01). However, N content in the faeces did not reflect overall CP and N intake, as the highest levels were seen in the control, non-xylanase-fed pigs and decreased linearly with increasing xylanase supplementation (*p* < 0.0001). Urine N was not affected by treatment, meaning that N digestibility and retention were also linearly as the levels of xylanase supplementation went up (*p* = 0.0224 digestibility, *p* = 0.0314 retention). Quadratic responses were not seen for N balance parameters.

### 3.3. Intestinal Morphology

The effects of xylanase supplementation on duodenal and jejunal morphology are summarised in Table 5. There were no significant differences between treatments on duodenal morphology. However, there was a significant linear relationship between increasing xylanase dose and villus height in the duodenum (*p* = 0.0480). In the jejunum, there was a significant linear relationship between jejunal villus height and xylanase supplementation (*p* < 0.0001), and a near-significant quadratic relationship (*p* = 0.0828). There was also a near significant linear relationship between jejunal crypt depth and xylanase supplementation (*p* = 0.0870), with crypt depth tending to increase with increasing levels of xylanase in the diet.

### 3.4. Faecal Volatile Fatty Acids and Ammonia Concentrations

The effects of xylanase on VFA concentrations in the faeces of weaning pigs at 35 days of age are summarised in Table 6. While there were limited significant differences between treatments, there were strong linear and quadratic relationships between increasing xylanase supplementation levels and propionate concentration. Propionate concentrations in the faeces had a significantly negative quadratic relationship (*p* = 0.0284) with increasing xylanase dose; this suggested that the highest levels of propionate would be recovered in the faeces of pigs fed approximately 55,000 U/kg. The relationship between xylanase and butyrate levels was less significant than that for propionate, and only the linear relationship was significant (*p* = 0.0161). No significant relationships were seen between xylanase dose and acetate, isobutyrate and isovalerate concentrations.

**Figure 1:**
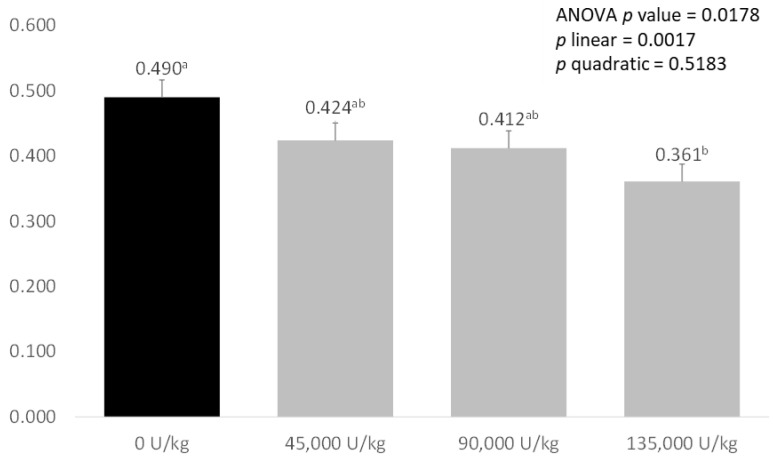
Effect of increasing levels of xylanase addition on ammonia emissions (mg/g) from the faeces of weaned piglets. Values are expressed as means of eight weaned pigs represented from each treatment. Values that significantly differ between treatments (with *p* < 0.05) are marked with different letter.

As shown in Figure 1, there was a significant effect of treatment on ammonia emissions from the faeces of weaned piglets (*p* = 0.0178). Faeces from piglets fed the control diets had the highest ammonia emissions, with 45,000 and 90,000 U/kg xylanase diets tending to reduce emissions. Faeces from pigs fed diets supplemented with 135,000 U/kg xylanase had significantly lower ammonia emissions compared with the control diet (0.490 mg/g CON vs. 0.361 mg/g XYL). There was a significant, negative linear relationship between faecal ammonia emissions and increasing xylanase dose (*p* = 0.017).

## 4. Discussion

Although the complete mechanism by which xylanase increases nutrient digestibility is not fully understood, the use of exogenous enzymes, including xylanase, to improve nutrient digestibility and retention is an established commercial practice. Torres-Pitarch et al. [14] found that, on average, the addition of xylanase to the diets of weaned piglets improved ADG by 28.1 g and G:F by 0.016 g/g. In the current study, the lowest level of supplementation with new xylanase from *Thermopolyspora flexuosa* and expressed in *Pichia pastoris* tended to improve ADG by 24.97 g and G:F by 0.031 g/g. Higher levels of supplementation were able to further improve growth performance, with significant improvements seen versus control at 90,000 U/kg. At 135,000 U/kg ADG and G:F were significantly improved compared to both the control and lower doses of xylanase, with increases compared to the control diet of 72.29 g ADG and 0.135 g/g G:F across the whole study. These improvements in performance followed a linear relationship with increasing xylanase dose. Many studies report classic non-linear responses to increasing levels of enzyme supplementation, with higher doses reporting limited further advantages [31,32,33,34]. The lack of quadratic response in the present study is in line with the findings of Kiarie and Petracek [35] and He et al. [10], who saw significant linear responses to xylanase submission but non-significant quadratic responses.

Most xylanases are able to improve the performance of wheat-based diets to a greater extent than of diets based on corn [36]; although corn accounts for nearly half of the dietary arabinoxylans found in a diet [37]. Corn-derived arabinoxylans are often poorly degraded due to their structure, the degree of interaction with other components, abundant phenolic cross-linkages, arabinose substitutions, and lignification [38]. However, the comparative efficacy on different dietary substrates is enzyme specific [39]. The xylanase used in the present study was able to improve the performance of both broilers and layers fed corn-based diets [18,19]. This can be possibly explained by two factors: an increase in feed efficiency through the release of encapsulated nutrients in the plant cell wall, and microbiome modulation via the prebiotic effect of the released xylo-oligosaccarides (XOS) from arabinoxylan hydrolysis. Improvements in the digestibility of starch and crude protein observed in both the present study and a previous study in broilers using the same xylanase could be related to the reduction in cage effect with the release of encapsulated nutrients such as starch and protein [18].

Traditionally, these performance improvements following xylanase supplementation were assumed to be driven by changes in gut viscosity [10] and nutrient digestibility. In the present study, supplementation with xylanase significantly improved the ATTD of dry matter, crude protein, ether extract, NDF, ADF, starch and gross energy. Gross energy was substantially increased by the lowest level of xylanase supplementation, jumping from 54.96% in the control diet to 68.4% with 45,000 U/kg xylanase (*p* < 0.05). Higher levels of xylanase supplementation resulted in further numerical increases, to a maximum of 78.76% with 135,000 U/kg xylanase. When looking at the efficiency of energy conversion, the increase in apparent energy digestibility is clear: pigs fed the control diet consumed 6019.16 kcal digestible energy (DE) per kilogram of body weight gain, with stepwise improvements at each level of xylanase supplementation (5689.61 kcal DE/kg BWG at 45,000 U/kg, 5438.46 kcal DE/kg BWG at 90,000 U/kg, 4825.36 kcal DE/kg BWG at 135,000 U/kg).

However, recent research has exposed a more complicated mode of action for supplemental xylanase than simple viscosity reduction and digestibility improvement. By breaking down the long arabinoxylan chains into shorter oligosaccharides, the substrate available for bacterial growth changes. This drives shifts in the microbial community in the gut, increasing VFA concentrations, reducing intestinal pH, and reducing inflammation. Combined with reductions in viscosity and reductions in nutrient caging by long fibre chains, this results in large and more resilient villi which promote better nutrient uptake across the gut barrier. In the present study, supplementation with xylanase significantly increased the levels of propionate in the faeces and tended to influence butyrate concentration. Other VFAs were not significantly affected by xylanase supplementation, in line with the results of Tsai et al. [1], who reported a limited effect of xylanase supplementation on faecal VFA concentrations in weaning pigs. O’Shea et al. [40] reported no effect of xylanase on individual VFA concentrations, though total faecal VFA concentration was significantly reduced with the addition of xylanase to growing pig diets. A similar lack of impact of xylanase addition on VFA concentrations was reported by Taylor et al. [41] and O’Connell et al. [42]. Tsai et al. suggested that the limited observed response may be due to the rapid absorption of VFAs in the intestine [43], with only 10% of VFA excreted in faeces [44]. It is possible that larger shifts in the VFA profile between treatments would be apparent in the analysis of ileal or caecal contents, compared to faeces, and this poses a future avenue of investigation. The quadratic response of propionate concentrations to xylanase addition is interesting, as it is unique amongst the VFA parameters measured in the present study.

Increasing levels of VFA may suggest a shift in the microbiome: Zhao et al. [22] reported piglet diets that promoted higher faecal concentrations of butyrate were linked to greater abundances of *Actinobacteria* and *Firmicutes*. Zhang et al. [43] reported a decreased abundance of *Bacteroidetes* and an increased abundance of *Firmicutes* following xylanase supplementation, while Sutton et al. [45], Luise et al. [21] and Gonzalez-Ortiz et al. [20] reported increased levels of *Lactobacilli*; similar responses have been seen following xylanase supplementation in poultry [18,19,46,47]. The genera involved in these shifts are associated with fibre-degrading mechanisms, breaking down complex polysaccharides and shorter-chain arabinoxylo-oligosaccharides, into lactic acid, hydrogen and SCFAs [48]. These products of fibre degradation are then consumed by Clostridium cluster XIV and other butyrate-producing bacteria in a mechanism known as cross-feeding [48]. The prebiotic mode of action of xylanase could also explain the improved diversity and altered microbiota ecology in the large intestine of pigs fed corn-based feed ingredients supplemented with xylanase [15,44]. Furthermore, there are increasing reports of the positive impact of xylanase supplementation on the markers of improved gastrointestinal health in swine with a reduction in finishing pig mortality [49], probably resulting from the modulation of microbial populations in the gut. Though not measured in the present study, the intestinal health changes resulting from xylanase supplementation are likely to improve the health of commercial swine herds and production sustainability.

These changes in bacterial populations and metabolic pathways may also explain the linear decrease in faecal ammonia emissions seen with increasing xylanase supplementation. Supplementation with 45,000 and 90,000 U/kg xylanase, led to large, near-significant reductions of, respectively, 13.5% and 15.9% in ammonia excretion, while supplementation with 135,000 U/kg xylanase significantly reduced faecal ammonia emissions (0.490 mg/g control vs. 0.361 mg/g 135,000 U/kg xylanase, −26.3%, *p* < 0.05). By contrast, McAlpine et al. [50] saw no significant effect when supplementing xylanase only however, when xylanase and protease were fed together, a significant reduction in ammonia emissions was observed. Reducing ammonia emissions is important both environmentally and socially for pig production, as reducing nitrogen excretion from commercial farms is a major target for producers, and reduction in smell from production facilities is important for farms close to residential areas.

## 5. Conclusions

In conclusion, supplementation with the xylanase molecule assessed in this study was able to improve growth and nutrient digestibility at the lowest dose (45,000 U/kg). The beneficial effects of xylanase addition increased with increasing dose, including improvements in G:F, nutrient utilisation, and villus height, whereas decreased propionate concentration and faecal ammonia emissions. These positive effects are likely driven both by the reduction of the caging effects of fibre, but also driven changes in VFA profile between treatments. This benefit may change in the intestinal microbial population of the weaned pigs. Further work should focus on elucidating the complementarity between these different modes of action.

## Figures and Tables

**Table 1:** Ingredient and nutrient composition (% as fed) of the basal diets given in the pre-starter (d0–14) and starter (d15–35) phases.

Raw Materials, %	Pre-Starter	Starter
Corn, 8% CP	34.45	34.29
Wheat	20.00	30.00
Broken rice	10.00	5.50
Full-fat soybean meal	8.00	3.50
Sweet whey	8.00	2.70
Soybean meal, 48% CP	5.00	13.10
Soy protein concentrate	5.00	1.00
Wheat bran	5.00	5.00
Rice bran oil	1.00	1.50
Limestone	0.61	0.92
Acid Lac ^1^	0.50	–
Monocalcium phosphate	0.30	0.45
Sodium chloride	0.40	0.35
L-lysine HCl	0.50	0.52
DL-methionine	0.24	0.21
L-threonine	0.20	0.20
L-valine	0.15	0.12
L-tryptophan	0.08	0.08
L-isoleucine	0.06	0.05
Phytase ^2^	0.01	0.01
Vitamin-mineral premix ^3^	0.50	0.50
Total	100.00	100.00
Nutrient composition, %		
Metabolisable energy, kcal/kg	3296.21	3244.49
Digestible energy, kcal/kg	3446.08	3467.15
Net energy, kcal/kg	2496.60	2482.61
Crude protein	17.35	17.03
Ca	0.61	0.71
Available P	0.40	0.39
Digestible Lys	1.11	1.09
Digestible Met + Cys	0.67	0.64
Digestible Thr	0.66	0.64
Digestible Ile	0.61	0.58
Ash	4.75	4.77
Crude fibre	2.59	2.67

^1^ Acidifier (Anhui Cofco Biochemical and Galactic Lactic Acid Co., Ltd., Bengbu, China); ^2^ Provided 500 FTU/kg product (Quantum Blue, AB Vista, Marlborough, UK); ^3^ Contains vitamin A, 4,000,000 (4,000,000) IU; vitamin D 600,000 (360,000) IU; vitamin E, 8 (2.5) g; vitamin K3, 0.40 (0.40) g; vitamin B1, 0.30 (0.25) g; vitamin B2, 1 (0.70) g; vitamin B6, 0.50 (0.40) g; vitamin B12, 4 (4) mg; niacin, 4 (3.6) g; choline chloride, 30 (19.77) g; calcium pantothenate, 3 (1.8) g; biotin, 10 (14) mg; folic acid, 0.10 (0.10) g; cobalt, 0.20 (0.09) g; copper, 40 (36) g; ferrous 36 (23) g; manganese, 16 (9.6) g; zinc, 20 (20) g; iodine, 0.20 (0.10) g; selenium, 0.02 (0.02) g; ethoxyquin, 10 (0.267) g; and silicon dioxide, 2 (10) g for prestarter (starter diet).

**Table 2:** Xylanase recovery in pelleted weaned piglet feeds compared to expected levels ^1^.

	Pre Starter, U/kg		Starter, U/kg	
Expected U/kg	Cold Mash	Pellet	Cold Mash	Pellet
0	n.d.^1^	n.d.	n.d.	n.d.
45,000	36,376	26,369	41,343	67,068
90,000	88,844	63,204	86,437	100,459
135,000	136,607	104,139	173,287	189,339

^1^ n.d. = not detected.

**Table 3:** Effect of increasing levels of dietary xylanase supplementation on piglet growth performance ^1^.

	Parameter	Xylanase, U/kg				SEM	*p*-Value		
		0	45,000	90,000	135,000		Treatment	Linear *	Quadratic **
d0-14	BW d0, kg	7.46	7.48	7.49	7.46	0.237	0.9997	0.9890	0.9227
	BW d14, kg	10.68	10.92	11.27	11.46	0.314	0.3114	0.0601	0.9389
	ADG, g	229.52	245.69	269.52	285.69	10.979	0.0051	0.0004	1.000
	ADFI, g	369.22	384.1	366.22	353.11	5.525	0.0044	0.0129	0.0182
	G:F	0.624	0.640	0.738	0.810	0.032	0.0007	<0.0001	0.3833
d15-35	BW d35, kg	19.14	20.03	20.26	21.67	0.381	0.0006	<0.0001	0.5053
	ADG, g	402.98	428.43	433.82	486.02	10.908	<0.0001	<0.0001	0.2498
	ADFI, g	756.78	762.67	746.56	743.00	3.357	0.0007	0.0010	0.1949
	G:F	0.532	0.568	0.572	0.653	0.014	<0.0001	<0.0001	0.1300
d0-35	ADG, g	333.60	358.57	364.86	405.89	7.525	<0.0001	<0.0001	0.3047
	ADFI, g	601.67	611.22	594.44	587.00	3.079	<0.0001	0.0003	0.0156
	G:F	0.557	0.588	0.612	0.692	0.013	<0.0001	<0.0001	0.0679
	Diarrhoea rate (%)	15.22 ^a^	9.78	7.89	5.89	1.628	0.0021	--	--

BW = body weight, ADG = average daily gain, ADFI = average daily feed intake, G:F gain-to-feed ratio, SEM = standard error of the means. ^1^ Each value shows least square means of nine replications of four weaned pigs (*n* = 36). * Linear effect (*p* < 0.05); ** Quadratic effect (*p* < 0.05). Values that significantly differ between treatments (with *p* < 0.05) are marked with different letter.

**Table 4:** Effect of increasing levels of dietary xylanase supplementation on apparent total tract digestibility of nutrients, energy, and nitrogen retention in weaned piglets ^1^.

	Parameters	Xylanase, U/kg				SEM	*p*-Value		
		0	45,000	90,000	135,000		Treatment	Linear *	Quadratic
Nutrient Digestibility, %	Dry matter	91.49	93.61	93.37	95.19	0.240	<0.0001	0.0002	0.7098
	Crude protein	87.29	90.61	90.68	93.33	0.505	0.0002	0.0002	0.6291
	Ether extract	82.60	88.90	87.24	89.45	1.034	0.0060	0.0106	0.1534
	Crude fibre	67.11	70.94	72.22	74.26	1.625	0.0721	0.0095	0.5781
	NDF	66.15	66.85	67.21	67.49	0.220	0.0128	0.0011	0.3436
	ADF	65.90	67.49	70.36	70.81	0.672	0.0024	0.0003	0.4306
	Gross energy	54.96	68.40	74.11	78.76	2.869	0.0020	0.0001	0.1448
	Starch	51.34	62.51	64.82	70.74	2.301	0.0022	0.0003	0.2952
N Balance	Crude protein intake	75.97	76.57	75.57	74.50	8.6 ×10^−9^	<0.0001	0.1256	0.6005
	N intake	12.15	12.25	12.09	11.92	--	--	--	--
	N-faeces	1.54	1.14	1.12	0.81	0.061	0.0002	0.0001	0.5668
	N-urine	5.63	5.33	5.57	4.63	0.346	0.2329	0.1107	0.3851
	N-retention	4.98	5.78	5.23	6.66	0.363	0.0470	0.0314	0.4786
	N-digestibility ^2^, %	40.96	47.15	43.90	55.04	3.007	0.0497	0.0224	0.4784

NDF = neutral detergent fibre, ADF = acid detergent fibre, SEM = standard error of the means. ^1^ A total of 12 crossbred pigs with an average BW of 10.22 kg were used in the digestibility trial (*n* = 12). ^2^ N retention = N intake (g)—faecal N (g)—urinary N (g) * linear effect (*p* < 0.05).

**Table 5:** Effect of increasing levels of dietary xylanase supplementation on duodenal and jejunal morphology of weaned piglets ^1^.

Parameters	Xylanase, U/kg				SEM	*p*-Value		
	0	45,000	90,000	135,000		Treatment	Linear *	Quadratic
Duodenum								
Villus height, µm	386.36	422.44	430.42	452.39	23.201	0.2669	0.0480	0.7598
Crypt depth, µm	313.23	322.93	327.90	327.48	18.849	0.9408	0.5685	0.7864
VH:CD	1.24	1.32	1.36	1.43	0.113	0.6878	0.2268	0.9745
Jejunum								
Villus height, µm	372.87	432.53	465.80	491.28	15.547	0.0001	<0.0001	0.0828
Crypt depth, µm	297.79	332.46	334.63	332.40	15.921	0.3177	0.0870	0.2496
VH:CD	1.26	1.34	1.42	1.49	0.090	0.3224	0.0618	0.9745

VH:CD = villus height/crypt depth ratio, SEM = standard error of the means. ^1^ Values are expressed as means of seven weaned pigs represented from each treatment (N = 28). * Linear effect (*p* < 0.05).

**Table 6:** Effect of increasing levels of dietary xylanase supplementation on volatile fatty acid concentrations in the faeces of weaned piglets ^1^.

Parameters	Xylanase, U/kg				SEM	*p*-Value		
	0	45,000	90,000	135,000		Treatment	Linear *	Quadratic **
Volatile Fatty Acids, mmol/L								
Acetate	17.79	16.89	19.55	18.56	2.629	0.9055	0.6715	0.9855
Propionate	8.55	9.16	14.40	10.59	0.933	0.0011	0.0364	0.0284
Butyrate	3.98	5.49	5.29	6.14	0.583	0.0971	0.0161	0.5785
Isobutyrate	1.79	2.29	1.00	2.13	0.328	0.0511	0.8753	0.4118
Isovalerate	2.64	2.03	2.01	1.97	0.456	0.6919	0.3167	0.5316
Total VFA	34.75	35.86	42.25	39.39	2.664	0.2102	0.1187	0.4689

VH:CD = total volatile fatty acids, SEM = standard error of the means. ^1^ Values are expressed as means of seven weaned pigs represented from each treatment (N = 28). * Linear effect (*p* < 0.05); ** Quadratic effect (*p* < 0.05).

## Data Availability

The data presented in this study are available on request from the corresponding author.
